# Effects of self-selected music on psychophysiological responses to goal-directed exercise in Parkinson's disease

**DOI:** 10.3389/fresc.2026.1778451

**Published:** 2026-04-14

**Authors:** Sophia L. Porrill, Jane B. Allendorfer, Alexandra M. Evancho, Christine C. Ferguson, Jenna M. LaChenaye, Haley M. Nguyen, Rebecca R. Rogers, Christopher G. Ballmann

**Affiliations:** 1 Department of Human Studies, University of Alabama at Birmingham, Birmingham, AL, United States; 2 Department of Neurology, University of Alabama at Birmingham, Birmingham, AL, United States; 3 Department of Physical Therapy, University of Alabama at Birmingham, Birmingham, AL, United States; 4 Department of Nutrition Sciences, University of Alabama at Birmingham, Birmingham, AL, United States; 5 Department of Family and Community Medicine, University of Alabama at Birmingham, Birmingham, AL, United States

**Keywords:** exercise, gait, motivation, music, neurodegeneration

## Abstract

Exercise is a cornerstone adjunct therapy for people with Parkinson's Disease (PwP) but tolerance is often impaired by non-motor processes including decreased motivation, psychological arousal, and physical effort allocation. Self-selected music (SSM) improves motivation and arousal state thereby enhancing physical effort and capacity during goal-directed exercise in healthy adults. Whether these effects translate to PwP is unclear. The purpose of this study was to measure the effects of SSM on psychophysiological responses and goal-directed exercise performance during a 6-minute walk test (6MWT) in PwP. In a counterbalanced crossover manner, PwP (*n* = 12) completed two 6MWT under different listening conditions: 1) White noise (WN) and 2) SSM. Participants walked as far as possible in six minutes while listening to the corresponding condition while heart rate, metabolic equivalents (METs), steps, and distance were monitored. After the completion of each 6MWT, motivation, psychological arousal, enjoyment, and rating of perceived exertion (RPE) were assessed. Findings show SSM music resulted in significantly higher heart rate (*p* = 0.004; d = 1.1), METs (*p* = 0.013; d = 0.9), total steps (*p* = 0.029; d = 0.8), and distance (*p* = 0.044; d = 0.6) compared to WN. Levels of psychological arousal (+62.6%; *p* = 0.001; d = 1.6), motivation (+94.1%; *p* < 0.001; d = 1.4), and enjoyment (*p* < 0.001; d = 2.0) were higher with SSM compared to WN. No differences in RPE were seen with SSM vs. WN (*p* = 0.309; d = 0.3). These preliminary data suggest SSM may aid in improving goal-based exercise performance and psychophysiological determinants of exercise capacity in PwP. Although larger sample sizes and more robust testing are warranted, SSM may be an effective ergogenic tool for PwP.

## 1 Introduction

Parkinson's disease (PD) results in pathological aging underpinned by dopaminergic insufficiency resulting in motor and non-motor symptoms. PD is one of the fastest-growing neurological diagnoses in aging populations globally with prevalence growing ∼2.5 times higher from years 1990 to 2016 (1). Currently, no cure for PD exists and many therapeutic strategies may lose efficacy, have high interindividual variability, or do not adequately address all symptoms (2). Regular exercise is a foundational therapy in the treatment of PD, improves motor and non-motor symptoms, and attenuates disease progression (3, 4). While motor dysfunction characteristic of PD progression can contribute to lower exercise ability, patient-reported barriers to the implementation of exercise interventions in PD have been largely linked to non-motor symptoms including losses in motivation, emotional dysfunction, and excessive fatigue (5). Thus, identifying feasible strategies to counteract non-motor barriers to exercise in PD is paramount to allow for the full realization of health benefits.

Listening to music, particularly self-selected music (SSM), has been shown to impart ergogenic effects and psychophysiological benefits during exercise. Indeed, copious amounts of evidence support benefits from SSM during exercise which may translate to neurological conditions (6). In healthy adults, previous evidence has shown that listening to SSM improves exercise capacity during endurance, high intensity interval, and resistance modes of exercise (7). SSM may also improve functional capacity and emotional state in older adults even in the presence of comorbidities (8, 9). The ergogenic effects of SSM are largely rooted in adaptative psychophysiological responses with non-motor origins. SSM reduces perceptions of fatigue and exertion through enhanced dissociation during exercise (10, 11). SSM increases arousal and neural activity linked to improved task engagement and effort (12, 13). Increased motivation with SSM may also lead to improved exercise outcomes through the enhancement of perceived task value and effort allocation (14). Thus, SSM may improve psychophysiological determinants of exercise capacity and contrast many of the dysfunctional non-motor processes from pathological aging which limit exercise in PD.

Use of music in rehabilitation has become more widely utilized in practice for individuals with PD through rhythmical auditory perception, motor planning, and movement behavior (15, 16). Using auditory cueing through music has been suggested to improve gait cadence, stride length, and walking speed in individuals with PD (15, 17). Music may also enhance premotor and motor planning that can aid in gait flow and motor symptom management. While promising, most investigations on music and PD have focused on motor symptoms as exercise barriers, leaving the potential benefits to non-motor processes relatively understudied (18). Given previous evidence showing benefits to non-motor psychophysiological processes during exercise in healthy populations, SSM may be an effective strategy to counteract non-motor exercise barriers in neurological conditions such as PD. Accordingly, the purpose of this pilot study was to identify psychophysiological and physical responses to goal-directed exercise while listening to SSM in people with PD. We hypothesized that compared to a noise control, SSM would result in improved exercise performance and psychophysiological determinants (i.e., motivation, enjoyment, arousal, perceived exertion) of exercise capacity during a 6-minute walk test in individuals with PD.

## 2 Methods

### 2.1 Participants

A convenience sample of individuals with idiopathic PD (*n* = 12; age = 67.0 yrs ± 8.6; height = 178.1 cm ± 8.8; body mass = 81.0 kg ± 9.7; age of diagnosis = 58.2 yrs ± 11.5) were recruited from the surrounding Birmingham, AL area. Participants were recruited from local PD exercise classes and through a participant list from our research group where participants consented to being contacted about future research studies. Inclusion criteria for participants included: formal diagnosis of idiopathic PD, Middle or old age onset of PD (at least 50 years of age), the ability to ambulate without assistive devices, stable medication regime for 4 weeks prior to participation, and no changes to treatment of PD in the last month. Our exclusion criteria consist of a non-PD related psychiatric disease (i.e., pre-diagnosis of PD), reports of falling in the last 6 weeks, and any reason a participant or their healthcare team believed their participation in the study could negatively impact their well-being. All participants provided written informed consent prior to participating in study procedures which were approved by the University of Alabama at Birmingham Institutional Review Board.

### 2.2 Self-selected music and control conditions

For the control condition, broadband (white) noise was used. White noise was chosen due to its flat spectral density and composition of random signals that span across the full range of human hearing (20–20,000 Hertz). Furthermore, white noise has been previously shown to be a neutral sound stimulus in previous investigations (19, 20). For the SSM condition, participants were instructed to pick a single musical composition from any genre that they would listen to if they “wanted to perform their best”. All music pieces were played on repeat and had to have a minimum tempo of 120 beats per minute that was confirmed using a music streaming (Spotify, Stockholm, Sweden) platform (7). Participants were instructed to pick a different musical piece until a song with appropriate tempo criteria was chosen. WN and SSM conditions were delivered using Bluetooth earbuds (Apple, Cupertino, CA, USA) at a self-selected comfortable volume (46). The mean tempo of selected songs was 135 bpm ± 10 and included genres such as rock n' roll, pop, heavy metal, rhythm and blues, and folk music.

### 2.3 Procedures

To assess responses to SSM during goal-directed exercise, participants completed two 6-minute walk tests (6MWT) under different counterbalanced conditions: 1) WN, 2) SSM. For the 6MWT, participants were instructed to walk as far as possible in the allotted time on a pre-defined 6MWT course inside a laboratory (21). The predefined course was a liner track 10 meters in length and approximately 3 meters wide where participants completed as many laps as possible. A chair was placed at the end of the course in case a participant became volitionally exhausted and needed to briefly recover during the walking assessment. However, a rest did not indicate abandonment of assessment, unless the participant indicated they would not be able to continue. Hemodynamic parameters (HR and blood pressure) were measured before and after each 6MWT to ensure safety during recovery. After participants completed the first 10 meters of walking on the 6MWT course, researchers recorded the duration and step count for the subsequent 10 meters to calculate walking velocities for analysis. To capture performance and psychophysiological outcomes during the 6MWT, a medical grade smart watch (EMBRACE PLUS; Empatica, inc., Milano, Italy) was worn on the wrist of the least affected side. The EMBRACE PLUS is equipped with advanced optical photoplethysmography (PPG), temperature sensors, and a multi-plane accelerometer and gyroscope (22, 23). Specifically, total steps, distance, HR, and metabolic equivalents (METs) were collected with an Epoch of 60 s. Following the completion of each 6MWT, participants completed visual analog scales to quantify feelings of motivation, enjoyment, and psychological arousal as previously described by Ballmann et al. and Rogers et al. (11, 24). Briefly, participants were asked to mark their perceptions for each outcome on a 100 mm line where zero indicated the absence of the feeling and one hundred indicated most extreme feelings. Measurements from zero to the point on the line where the participant marked were recorded and used for data analysis. Furthermore, rating of perceived exertion (RPE) was obtained at the cessation of walking exercise on a 1–10 scale where participants were asked to rate how hard they felt the exercise was ranging from 1 “easy” to 10 “maximal exertion” (11, 25). 6MWT for each condition were separated by 20 min of rest.

### 2.4 Data analysis

All data were analyzed using open-access statistical software (Jamovi, Version 0.9). Normality of data was determined using the Shapiro–Wilk method and are shown as group means and individual performances. For all outcomes, comparisons between conditions were analyzed using a paired samples t-test. Effect size estimations between means were calculated via Cohen's D and interpreted as 0.2-small, 0.5-, moderate, 0.8-large (26). Significance was set *a priori* at *p* ≤ 0.05.

## 3 Results

### 3.1 Performance outcomes

**Figure 1:**
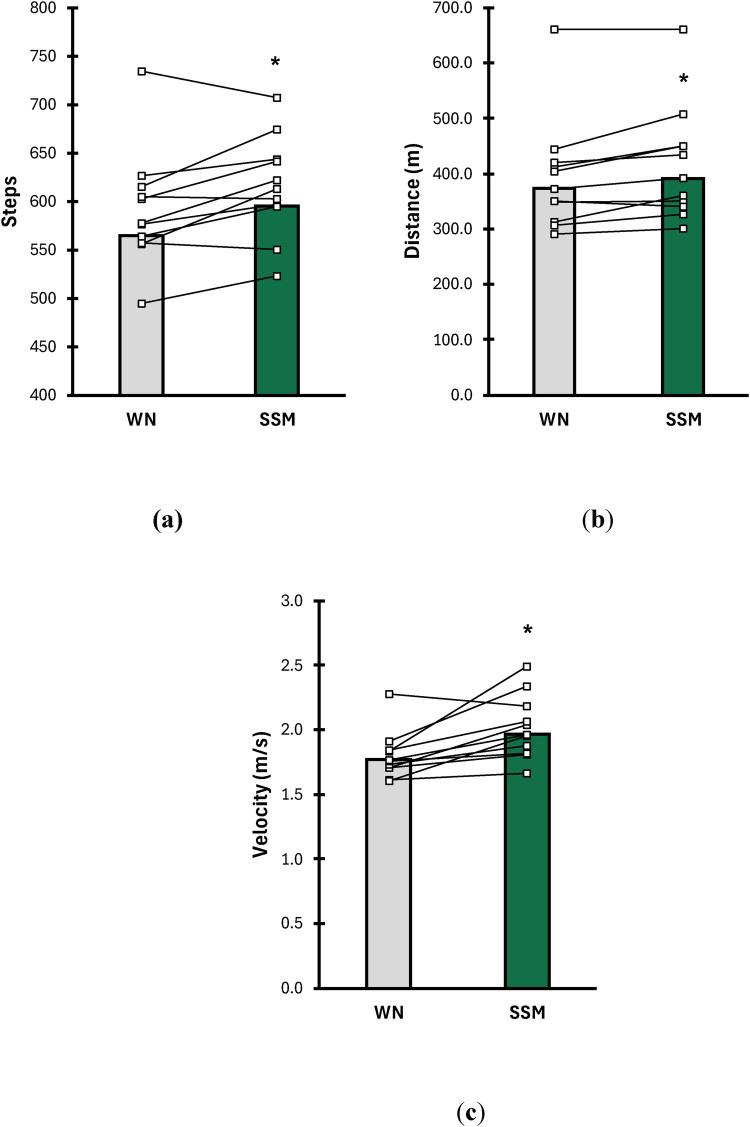
Comparisons of **(a)** total steps (steps), **(b)** total distance **(**m**)**, and **(c)** walking velocity (m × s^−1^) between WN (grey bars) and SSM (green bars) during a 6-minute walk test (6MWT). Data are presented as means (bars) and individual responses (lines) for each condition. * indicates means of SSM are significantly different from WN (*p* ≤ 0.05).

Results of total distance (meters), total steps (steps), and walking velocity (m × s^−1^) are shown in Figure 1. For total steps (Figure 1a), participants accumulated significantly more steps during the SSM condition vs. WN (WN = 564 steps ± 105, SSM = 595 steps ± 79; *p* = 0.016; d = 0.81). Furthermore, participants accumulated a greater total distance (Figure 1b) during the SSM condition vs. WN (WN = 372 m ± 125, SSM = 392 m ± 123; *p* = 0.044; d = 0.66). For walking velocity (Figure 1c), participants showed significantly higher walking velocity during the SSM condition compared to WN (WN = 1.7 m × s^−1^ ± 0.2, SSM = 1.96 m × s^−1^ ± 0.3; *p* = 0.009; d = 0.91).

### 3.2 Psychophysiological outcomes

**Figure 2:**
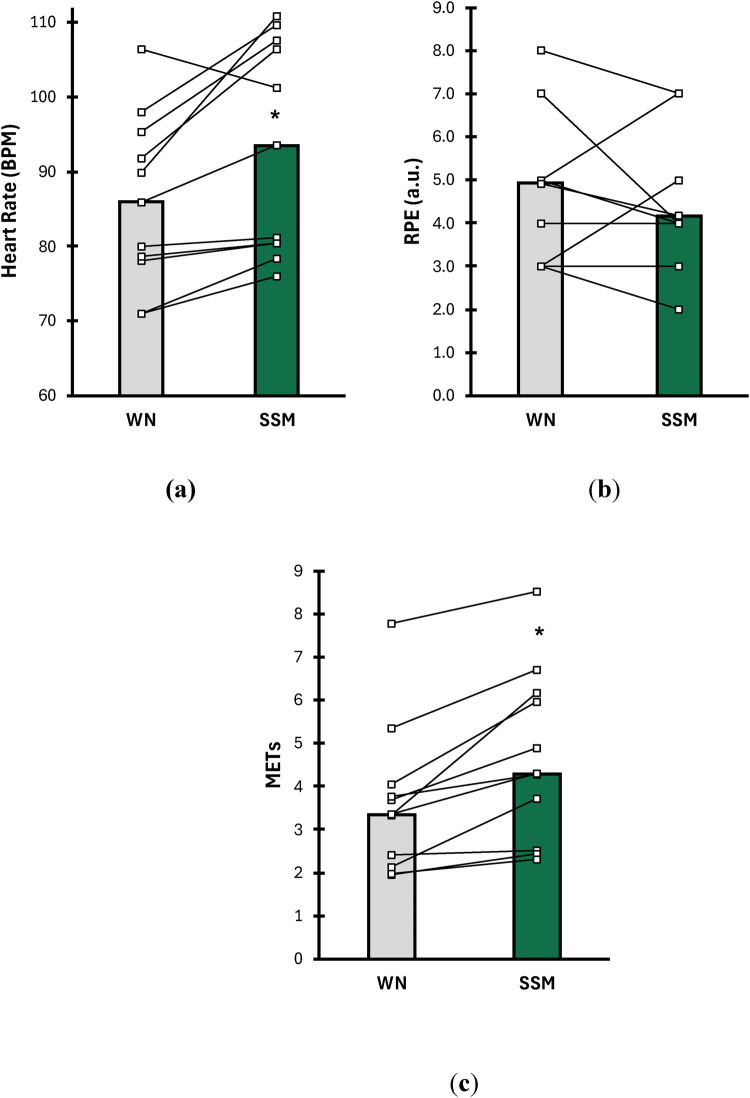
Comparisons of **(a)** mean heart rate (HR; beats per minute), **(b)** rating of perceived exertion (1–10 scale), and **(c)** metabolic equivalent of task (measure of energy used during physical activity) between WN (grey bars) and SSM (green bars) during a 6-minute walk test (6MWT). Data are presented as means (bars) and individual responses (lines) for each condition.* indicates means of SSM are significantly different from WN (*p* ≤ 0.05).

Results of mean heart rate (bpm), rating of perceived exertion (RPE; 1–10 scale), and metabolic equivalent of task (METs; a.u.) are shown in Figure 2. For mean heart rate (Figure 2a), SSM resulted in significantly higher heart rate during the 6MWT compared to WN (WN = 86 bpm ± 13, SSM = 94 bpm ± 15; *p* = 0.004; d = 1.05). Rating of perceived exertion (RPE) (Figure 2b) was unaffected regardless of condition (WN = 4.9 a.u. ± 2.2, SSM = 4.1 a.u. ± 1.6; *p* = 0.250; d = 0.35). However, METs (Figure 2c) were significantly higher during the 6MWT during the SSM condition compared to WN (WN = 3.4 a.u. ± 1.8, SSM = 4.3 a.u. ± 2.1; *p* = 0.003; d = 1.07).

**Figure 3:**
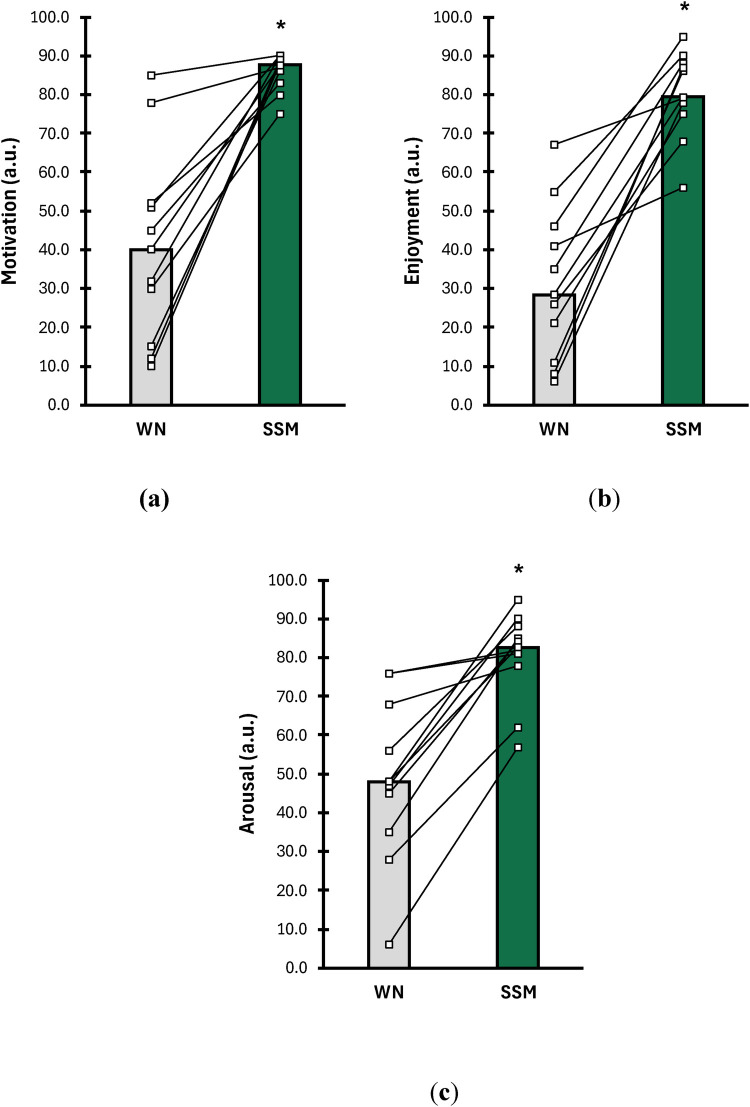
Comparison of **(a)** motivation (arbitrary units; a.u.), **(b)** enjoyment (arbitrary units; a.u.), and **(c)** arousal (arbitrary units; a.u.) between WN (grey bars) and SSM (green bars) during a 6-minute walk test (6MWT). Data are presented as means (bars) and individual responses (lines) for each condition. * indicates means of SSM are significantly different from WN (*p* ≤ 0.05).

Results of motivation (arbitrary units; a.u.), enjoyment (arbitrary units; a.u.), and arousal (arbitrary units; a.u.) are shown in Figure 3. Motivation (Figure 3a) was significantly higher with SSM compared to WN during the 6MWT (WN = 40 a.u. ± 27, SSM = 88 a.u. ± 6; *p* < 0.001; d = 1.73). Furthermore, enjoyment (Figure 3b) was significantly higher with SSM vs. WN during the 6MWT (WN = 28 a.u. ± 20, SSM = 79 a.u. ± 11; *p* < 0.001; d = 2.30). Lastly, psychological arousal (Figure 3c) was significantly higher with SSM compared to WN during the 6MWT (WN = 48 a.u. ± 20, SSM = 83 a.u. ± 12; *p* < 0.001; d = 1.92).

## 4 Discussion

Exercise is a cornerstone therapy for individuals with PD that improves symptomology and attenuates disease progression (27). However, adherence and tolerance to exercise regimens are often thwarted by motor and non-motor symptoms (28). Listening to music during walking has been shown to improve gait and alleviate some motor-based exercise barriers (29). However, the effects of SSM on non-motor psychophysiological responses during exercise in PD are unknown. Thus, the purpose of this study was to investigate the effects of SSM on psychophysiological responses during walking exercise related outcomes in people with PD. Current findings show that in people with PD, listening to SSM during a 6MWT resulted in increased distance, steps, walking velocity, HR, and METs compared to WN despite unaltered RPE levels. Furthermore, perceptions of motivation, enjoyment, and psychological arousal were higher with SSM compared to WN. While underlying mechanisms are not fully clear at this time, this study shows preliminary evidence that SSM induces positive psychophysiological benefits and improves walking performance in people with PD which could hold important implications for the attainment of exercise benefits in this population.

Current findings show that listening to SSM enhanced walking performance metrics in individuals with PD compared to a noise control. This supports previous findings showing improvements in gait and motor function with music during rehabilitation in individuals with PD (30, 31). Listening to music may induce gait synchronization where step timing aligns to the beat or tempo of the stimulus (30). Rhythmic auditory stimuli, like music, may aid with gross motor skills, posture, and locomotion through alterations of reticulospinal activity and synchronizing muscle activation to musical patterns (32). Although not measured currently, these effects may be altered by familiarity and pleasantness of music. For example, Shin Park et al. showed that walking with familiar music improved stride and arm swing amplitude in individuals with PD compared to unfamiliar music (33). De Bartolo et al. also showed genre-dependent effects of music on gait parameters in individuals with PD (34). Increases in gait amplitude have been suggested to have a strong relationship with degree of perceived music pleasantness in individuals with PD (35). It has been previously postulated that pleasant music results in greater dopamine release within the striatum leading to additional stimulation for movement and physical effort (35, 36). While not confirmed currently leaving mechanisms largely speculative, current findings of increased walking capacity with SSM may support this and suggest a need for further mechanistic study into the benefits of SSM in PD.

Importantly, markers of exercise intensity (e.g., HR, METs) were notably higher during walking with SSM vs. a noise control. This may be reflective of the increases in work rate achieved or SSM-induced enhancement of physiological stimulation. Indeed, previous evidence has shown that listening to SSM increases physiological markers of stimulation and arousal including heart rate and respiratory rate (37). Interestingly, current data show that physiological markers of intensity were elevated without changes in RPE indicating that although participants maintained higher intensity during the walking bout with SSM, perception of work rate was unaltered. SSM has been well established to induce dissociative effects during exercise and lowers RPE during physical effort (7). From a practical standpoint, SSM-induced increases in exercise intensity may result in greater adaptation and without changes in perception of intensity, may lead to improvements in exercise tolerance and adherence in individuals with PD. Longitudinal studies investigating the potential benefits of SSM on exercise tolerance and adherence in individuals with PD are needed for the potential realization of therapeutic benefits.

Importantly, current improvements in walking outcomes were accompanied by potent enhancement of psychological determinants of exercise capacity. Improvements in psychological arousal, motivation, and enjoyment during exercise have been repeatedly reported with SSM in other populations. Motivational deficits, emotional dysfunction, and low energy levels have been previously identified as primary barriers to the engagement of exercise and physical activity in PD (38, 39). Current findings suggest the use of SSM may aid in counteracting these barriers by imparting psychological benefits. While underpinning mechanisms for these changes remain unclear, SSM may stimulate functioning neurons to increase dopamine and neurotransmitter release important for motivation and arousal state (40–42). Furthermore, it has been previously suggested that exercise may result in more sustained activity of dopamine in neural synapses which could be potentiated further by SSM (43, 44). Enhancement of motivation and arousal from SSM has been linked to increased allocation of effort and physical ability largely through a “psyching up” effect thereby leading to enhanced performance (7). SSM has also been previously shown to enhance emotional affect which may improve mood and enjoyment during exercise (7). Increases in arousal through heightened autonomic activity may also counteract dysfunctional cardiovascular responses to exercise in PD as evidenced currently by concomitant increases in psychological arousal and higher HRs (45). Overall, SSM appears to impart ergogenic effects for people with PD that may be mediated through improved psychological responses to exercise. Future studies should examine possible neural mechanisms underlying these acute changes and establish how they may improve exercise tolerance and adherence in people with PD.

## 5 Limitations and conclusion

While the current study provides novel information regarding the use of SSM as an effective tool to improve motivational and arousal state thereby increasing walking ability in people PD, there were several limitations. First, the sample size for this study was relatively small. Therefore, investigations with larger sample size will be necessary to translate these findings to the greater PD patient community. Also, the current study did not include healthy controls as a comparison group. Therefore, it is currently unknown if SSM is effective simply as a factor to counteract age-related declines in exercise or if results are specific to the pathology of PD. Another limitation of the current study is the possibility of a placebo effect. Since participants were instructed to self-select music that would help them “perform their best”, it is possible that this may have modified expectations and behavior during testing thereby enhancing performance. As previously discussed, it is plausible that SSM resulted in auditory-motor synchronization. However, the current study design limits conclusions due to omission of methods capable of determining the precise contribution of tempo-matched movement leaving this point largely speculative from current data alone. Lastly, only one mode of brief exercise in the 6MWT was used for assessment. Based on how participants rated their perceived exertion during each 6MWT, the 6MWT was light intensity exercise. Therefore, it remains unknown if these findings translate to other modes or intensities of exercise. Future studies should employ different forms and intensities of exercise to understand the implications of using SSM during exercise in people with PD. Although the mechanistic underpinnings of current findings of SSM on exercise in PD remain incomplete, this study shows that SSM may be an effective tool for to improve psychophysiological responses and goal-directed exercise performance in people with PD.

## Data Availability

The original contributions presented in the study are included in the article/Supplementary Material, further inquiries can be directed to the corresponding author.
